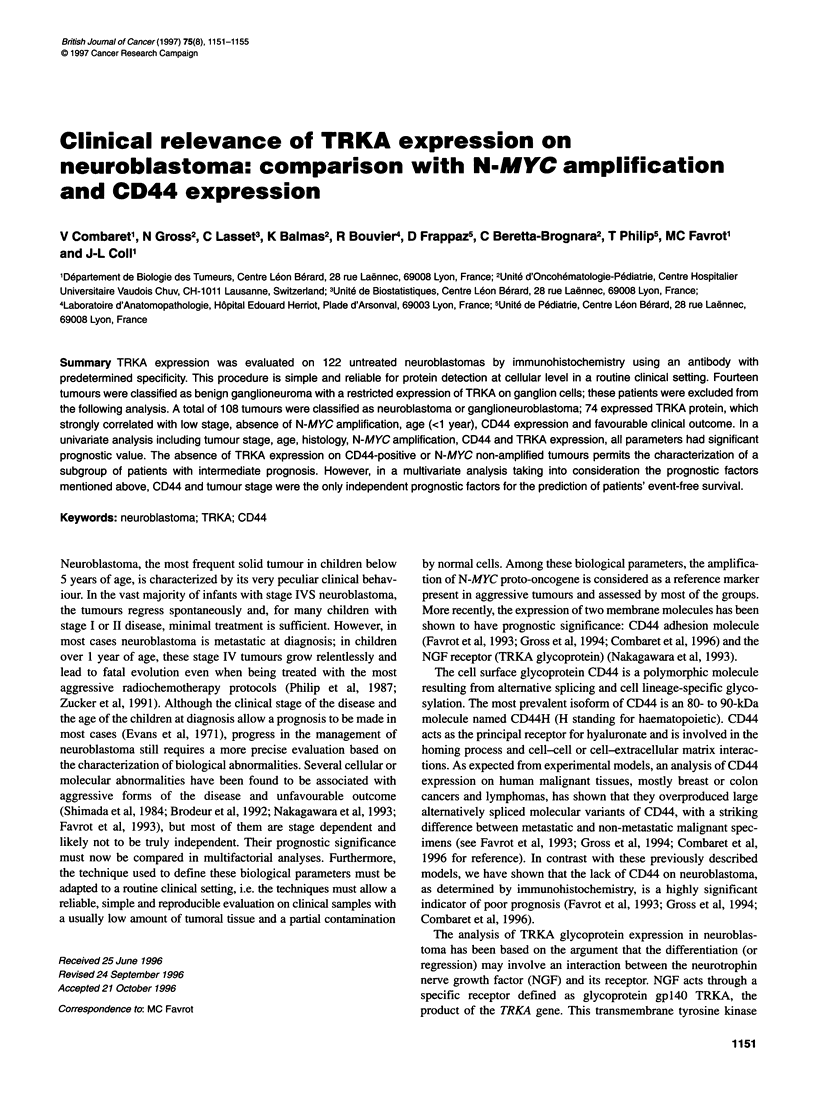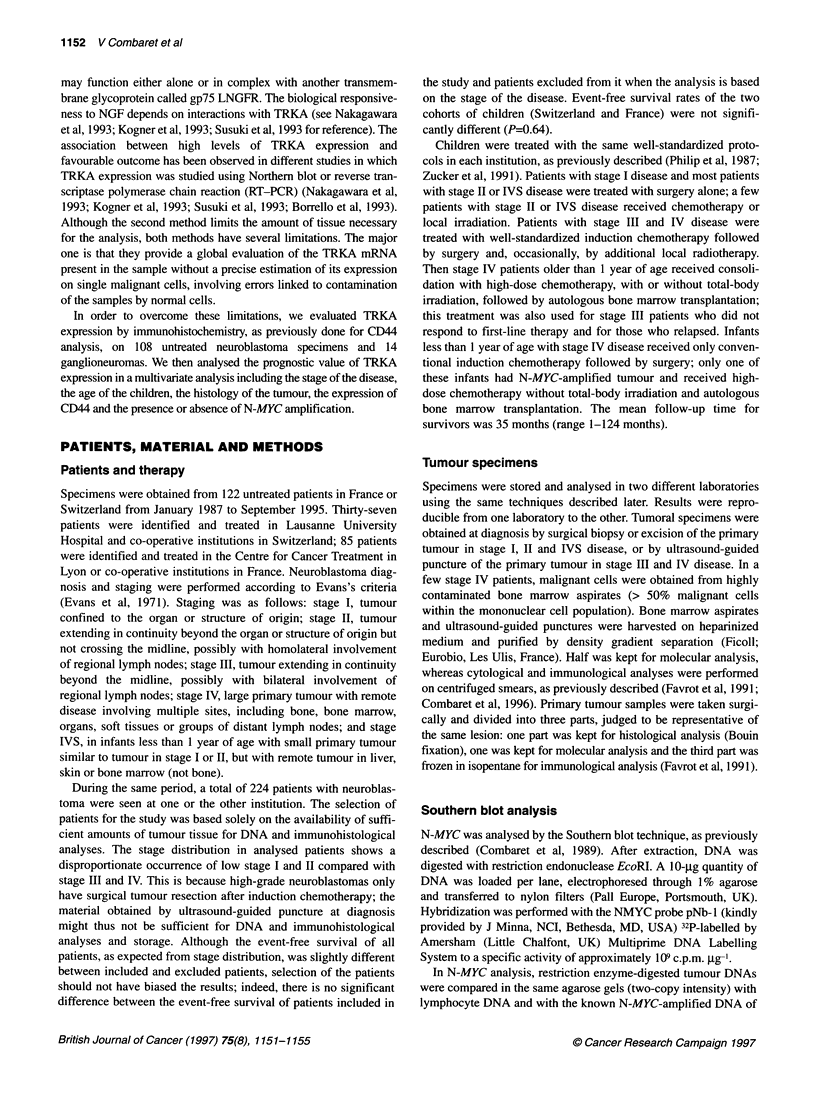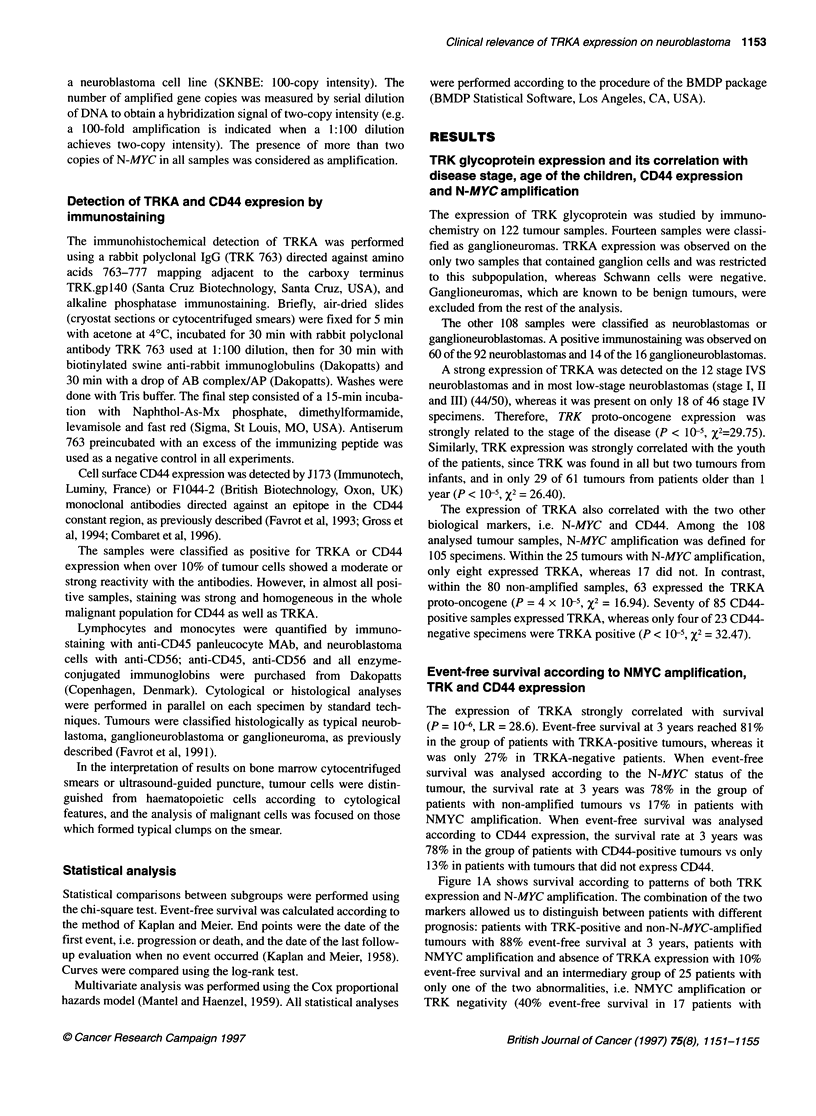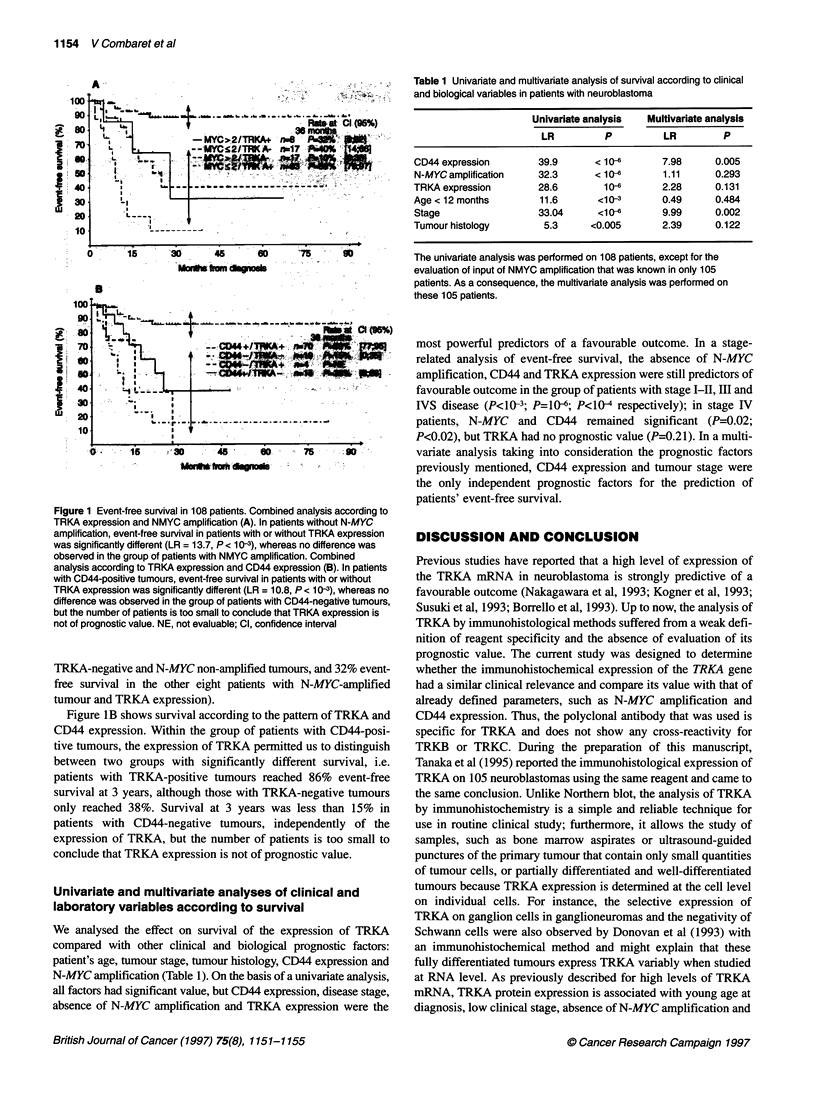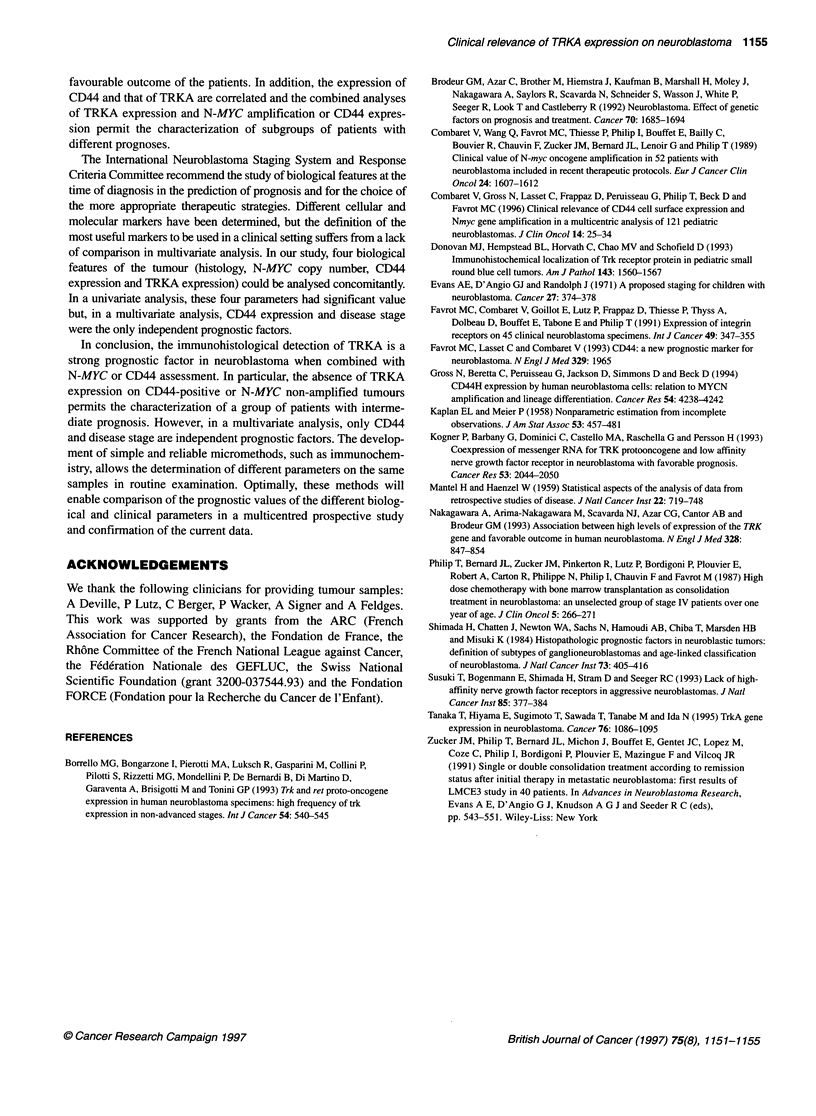# Clinical relevance of TRKA expression on neuroblastoma: comparison with N-MYC amplification and CD44 expression.

**DOI:** 10.1038/bjc.1997.198

**Published:** 1997

**Authors:** V. Combaret, N. Gross, C. Lasset, K. Balmas, R. Bouvier, D. Frappaz, C. Beretta-Brognara, T. Philip, M. C. Favrot, J. L. Coll

**Affiliations:** DÃ©partement de Biologie des Tumeurs, Centre LÃ©on BÃ©rard, Lyon, France.

## Abstract

TRKA expression was evaluated on 122 untreated neuroblastomas by immunohistochemistry using an antibody with predetermined specificity. This procedure is simple and reliable for protein detection at cellular level in a routine clinical setting. Fourteen tumours were classified as benign ganglioneuroma with a restricted expression of TRKA on ganglion cells; these patients were excluded from the following analysis. A total of 108 tumours were classified as neuroblastoma or ganglioneuroblastoma; 74 expressed TRKA protein, which strongly correlated with low stage, absence of N-MYC amplification, age (<1 year), CD44 expression and favourable clinical outcome. In a univariate analysis including tumour stage, age, histology, N-MYC amplification, CD44 and TRKA expression, all parameters had significant prognostic value. The absence of TRKA expression on CD44-positive or N-MYC non-amplified tumours permits the characterization of a subgroup of patients with intermediate prognosis. However, in a multivariate analysis taking into consideration the prognostic factors mentioned above, CD44 and tumour stage were the only independent prognostic factors for the prediction of patients' event-free survival.


British Journal of Cancer (1997) 75(8), 1151–1155
© 1997 Cancer Research Campaign

### Clinical relevance of TRKA expression on neuroblastoma: comparison with N-*MYC* amplification and CD44 expression

**V Combaret[1], N Gross[2], C Lasset[3], K Balmas[2], R Bouvier[4], D Frappaz[5], C Beretta-Brognara[2], T Philip[5], MC Favrot[1] and J-L Coll[1]**

[1]Département de Biologie des Tumeurs, Centre Léon Bérard, 28 rue Laënnec, 69008 Lyon, France; [2]Unité d'Oncohématologie-Pédiatrie, Centre Hospitalier Universitaire Vaudois Chuv, CH-1011 Lausanne, Switzerland; [3]Unité de Biostatistiques, Centre Léon Bérard, 28 rue Laënnec, 69008 Lyon, France; [4]Laboratoire d'Anatomopathologie, Hôpital Edouard Herriot, Plade d'Arsonval, 69003 Lyon, France; [5]Unité de Pédiatrie, Centre Léon Bérard, 28 rue Laënnec, 69008 Lyon, France

**Summary** TRKA expression was evaluated on 122 untreated neuroblastomas by immunohistochemistry using an antibody with predetermined specificity. This procedure is simple and reliable for protein detection at cellular level in a routine clinical setting. Fourteen tumours were classified as benign ganglioneuroma with a restricted expression of TRKA on ganglion cells; these patients were excluded from the following analysis. A total of 108 tumours were classified as neuroblastoma or ganglioneuroblastoma; 74 expressed TRKA protein, which strongly correlated with low stage, absence of N-*MYC* amplification, age (<1 year), CD44 expression and favourable clinical outcome. In a univariate analysis including tumour stage, age, histology, N-*MYC* amplification, CD44 and TRKA expression, all parameters had significant prognostic value. The absence of TRKA expression on CD44-positive or N-*MYC* non-amplified tumours permits the characterization of a subgroup of patients with intermediate prognosis. However, in a multivariate analysis taking into consideration the prognostic factors mentioned above, CD44 and tumour stage were the only independent prognostic factors for the prediction of patients' event-free survival.

**Keywords:** neuroblastoma; TRKA; CD44

Neuroblastoma, the most frequent solid tumour in children below 5 years of age, is characterized by its very peculiar clinical behaviour. In the vast majority of infants with stage IVS neuroblastoma, the tumours regress spontaneously and, for many children with stage I or II disease, minimal treatment is sufficient. However, in most cases neuroblastoma is metastatic at diagnosis; in children over 1 year of age, these stage IV tumours grow relentlessly and lead to fatal evolution even when being treated with the most aggressive radiochemotherapy protocols (Philip et al, 1987; Zucker et al, 1991). Although the clinical stage of the disease and the age of the children at diagnosis allow a prognosis to be made in most cases (Evans et al, 1971), progress in the management of neuroblastoma still requires a more precise evaluation based on the characterization of biological abnormalities. Several cellular or molecular abnormalities have been found to be associated with aggressive forms of the disease and unfavourable outcome (Shimada et al, 1984; Brodeur et al, 1992; Nakagawara et al, 1993; Favrot et al, 1993), but most of them are stage dependent and likely not to be truly independent. Their prognostic significance must now be compared in multifactorial analyses. Furthermore, the technique used to define these biological parameters must be adapted to a routine clinical setting, i.e. the techniques must allow a reliable, simple and reproducible evaluation on clinical samples with a usually low amount of tumoral tissue and a partial contamination by normal cells. Among these biological parameters, the amplification of N-*MYC* proto-oncogene is considered as a reference marker present in aggressive tumours and assessed by most of the groups. More recently, the expression of two membrane molecules has been shown to have prognostic significance: CD44 adhesion molecule (Favrot et al, 1993; Gross et al, 1994; Combaret et al, 1996) and the NGF receptor (TRKA glycoprotein) (Nakagawara et al, 1993).

The cell surface glycoprotein CD44 is a polymorphic molecule resulting from alternative splicing and cell lineage-specific glycosylation. The most prevalent isoform of CD44 is an 80- to 90-kDa molecule named CD44H (H standing for haematopoietic). CD44 acts as the principal receptor for hyaluronate and is involved in the homing process and cell–cell or cell–extracellular matrix interactions. As expected from experimental models, an analysis of CD44 expression on human malignant tissues, mostly breast or colon cancers and lymphomas, has shown that they overproduced large alternatively spliced molecular variants of CD44, with a striking difference between metastatic and non-metastatic malignant specimens (see Favrot et al, 1993; Gross et al, 1994; Combaret et al, 1996 for reference). In contrast with these previously described models, we have shown that the lack of CD44 on neuroblastoma, as determined by immunohistochemistry, is a highly significant indicator of poor prognosis (Favrot et al, 1993; Gross et al, 1994; Combaret et al, 1996).

The analysis of TRKA glycoprotein expression in neuroblastoma has been based on the argument that the differentiation (or regression) may involve an interaction between the neurotrophin nerve growth factor (NGF) and its receptor. NGF acts through a specific receptor defined as glycoprotein gp140 TRKA, the product of the *TRKA* gene. This transmembrane tyrosine kinase

*Received 25 June 1996*
*Revised 24 September 1996*
*Accepted 21 October 1996*

*Correspondence to:* MC Favrot may function either alone or in complex with another transmembrane glycoprotein called gp75 LNGFR. The biological responsiveness to NGF depends on interactions with TRKA (see Nakagawara et al, 1993; Kogner et al, 1993; Susuki et al, 1993 for reference). The association between high levels of TRKA expression and favourable outcome has been observed in different studies in which TRKA expression was studied using Northern blot or reverse transcriptase polymerase chain reaction (RT–PCR) (Nakagawara et al, 1993; Kogner et al, 1993; Susuki et al, 1993; Borrello et al, 1993). Although the second method limits the amount of tissue necessary for the analysis, both methods have several limitations. The major one is that they provide a global evaluation of the TRKA mRNA present in the sample without a precise estimation of its expression on single malignant cells, involving errors linked to contamination of the samples by normal cells.

In order to overcome these limitations, we evaluated TRKA expression by immunohistochemistry, as previously done for CD44 analysis, on 108 untreated neuroblastoma specimens and 14 ganglioneuromas. We then analysed the prognostic value of TRKA expression in a multivariate analysis including the stage of the disease, the age of the children, the histology of the tumour, the expression of CD44 and the presence or absence of N-*MYC* amplification.

### PATIENTS, MATERIAL AND METHODS

### Patients and therapy

Specimens were obtained from 122 untreated patients in France or Switzerland from January 1987 to September 1995. Thirty-seven patients were identified and treated in Lausanne University Hospital and co-operative institutions in Switzerland; 85 patients were identified and treated in the Centre for Cancer Treatment in Lyon or co-operative institutions in France. Neuroblastoma diagnosis and staging were performed according to Evans's criteria (Evans et al, 1971). Staging was as follows: stage I, tumour confined to the organ or structure of origin; stage II, tumour extending in continuity beyond the organ or structure of origin but not crossing the midline, possibly with homolateral involvement of regional lymph nodes; stage III, tumour extending in continuity beyond the midline, possibly with bilateral involvement of regional lymph nodes; stage IV, large primary tumour with remote disease involving multiple sites, including bone, bone marrow, organs, soft tissues or groups of distant lymph nodes; and stage IVS, in infants less than 1 year of age with small primary tumour similar to tumour in stage I or II, but with remote tumour in liver, skin or bone marrow (not bone).

During the same period, a total of 224 patients with neuroblastoma were seen at one or the other institution. The selection of patients for the study was based solely on the availability of sufficient amounts of tumour tissue for DNA and immunohistological analyses. The stage distribution in analysed patients shows a disproportionate occurrence of low stage I and II compared with stage III and IV. This is because high-grade neuroblastomas only have surgical tumour resection after induction chemotherapy; the material obtained by ultrasound-guided puncture at diagnosis might thus not be sufficient for DNA and immunohistological analyses and storage. Although the event-free survival of all patients, as expected from stage distribution, was slightly different between included and excluded patients, selection of the patients should not have biased the results; indeed, there is no significant difference between the event-free survival of patients included in the study and patients excluded from it when the analysis is based on the stage of the disease. Event-free survival rates of the two cohorts of children (Switzerland and France) were not significantly different ($P$=0.64).

Children were treated with the same well-standardized protocols in each institution, as previously described (Philip et al, 1987; Zucker et al, 1991). Patients with stage I disease and most patients with stage II or IVS disease were treated with surgery alone; a few patients with stage II or IVS disease received chemotherapy or local irradiation. Patients with stage III and IV disease were treated with well-standardized induction chemotherapy followed by surgery and, occasionally, by additional local radiotherapy. Then stage IV patients older than 1 year of age received consolidation with high-dose chemotherapy, with or without total-body irradiation, followed by autologous bone marrow transplantation; this treatment was also used for stage III patients who did not respond to first-line therapy and for those who relapsed. Infants less than 1 year of age with stage IV disease received only conventional induction chemotherapy followed by surgery; only one of these infants had N-*MYC*-amplified tumour and received high-dose chemotherapy without total-body irradiation and autologous bone marrow transplantation. The mean follow-up time for survivors was 35 months (range 1–124 months).

### Tumour specimens

Specimens were stored and analysed in two different laboratories using the same techniques described later. Results were reproducible from one laboratory to the other. Tumoral specimens were obtained at diagnosis by surgical biopsy or excision of the primary tumour in stage I, II and IVS disease, or by ultrasound-guided puncture of the primary tumour in stage III and IV disease. In a few stage IV patients, malignant cells were obtained from highly contaminated bone marrow aspirates (> 50% malignant cells within the mononuclear cell population). Bone marrow aspirates and ultrasound-guided punctures were harvested on heparinized medium and purified by density gradient separation (Ficoll; Eurobio, Les Ulis, France). Half was kept for molecular analysis, whereas cytological and immunological analyses were performed on centrifuged smears, as previously described (Favrot et al, 1991; Combaret et al, 1996). Primary tumour samples were taken surgically and divided into three parts, judged to be representative of the same lesion: one part was kept for histological analysis (Bouin fixation), one was kept for molecular analysis and the third part was frozen in isopentane for immunological analysis (Favrot et al, 1991).

### Southern blot analysis

N-*MYC* was analysed by the Southern blot technique, as previously described (Combaret et al, 1989). After extraction, DNA was digested with restriction endonuclease *Eco*RI. A 10-μg quantity of DNA was loaded per lane, electrophoresed through 1% agarose and transferred to nylon filters (Pall Europe, Portsmouth, UK). Hybridization was performed with the NMYC probe pNb-1 (kindly provided by J Minna, NCI, Bethesda, MD, USA) $^{32}$P-labelled by Amersham (Little Chalfont, UK) Multiprime DNA Labelling System to a specific activity of approximately $10^9$ c.p.m. $\mu g^{-1}$.

In N-*MYC* analysis, restriction enzyme-digested tumour DNAs were compared in the same agarose gels (two-copy intensity) with lymphocyte DNA and with the known N-*MYC*-amplified DNA of

© Cancer Research Campaign 1997

a neuroblastoma cell line (SKNBE: 100-copy intensity). The number of amplified gene copies was measured by serial dilution of DNA to obtain a hybridization signal of two-copy intensity (e.g. a 100-fold amplification is indicated when a 1:100 dilution achieves two-copy intensity). The presence of more than two copies of N-*MYC* in all samples was considered as amplification.

### Detection of TRKA and CD44 expresion by immunostaining

The immunohistochemical detection of TRKA was performed using a rabbit polyclonal IgG (TRK 763) directed against amino acids 763–777 mapping adjacent to the carboxy terminus TRK.gp140 (Santa Cruz Biotechnology, Santa Cruz, USA), and alkaline phosphatase immunostaining. Briefly, air-dried slides (cryostat sections or cytocentrifuged smears) were fixed for 5 min with acetone at 4°C, incubated for 30 min with rabbit polyclonal antibody TRK 763 used at 1:100 dilution, then for 30 min with biotinylated swine anti-rabbit immunoglobulins (Dakopatts) and 30 min with a drop of AB complex/AP (Dakopatts). Washes were done with Tris buffer. The final step consisted of a 15-min incubation with Naphthol-As-Mx phosphate, dimethylformamide, levamisole and fast red (Sigma, St Louis, MO, USA). Antiserum 763 preincubated with an excess of the immunizing peptide was used as a negative control in all experiments.

Cell surface CD44 expression was detected by J173 (Immunotech, Luminy, France) or F1044-2 (British Biotechnology, Oxon, UK) monoclonal antibodies directed against an epitope in the CD44 constant region, as previously described (Favrot et al, 1993; Gross et al, 1994; Combaret et al, 1996).

The samples were classified as positive for TRKA or CD44 expression when over 10% of tumour cells showed a moderate or strong reactivity with the antibodies. However, in almost all positive samples, staining was strong and homogeneous in the whole malignant population for CD44 as well as TRKA.

Lymphocytes and monocytes were quantified by immunostaining with anti-CD45 panleucocyte MAb, and neuroblastoma cells with anti-CD56; anti-CD45, anti-CD56 and all enzyme-conjugated immunoglobins were purchased from Dakopatts (Copenhagen, Denmark). Cytological or histological analyses were performed in parallel on each specimen by standard techniques. Tumours were classified histologically as typical neuroblastoma, ganglioneuroblastoma or ganglioneuroma, as previously described (Favrot et al, 1991).

In the interpretation of results on bone marrow cytocentrifuged smears or ultrasound-guided puncture, tumour cells were distinguished from haematopoietic cells according to cytological features, and the analysis of malignant cells was focused on those which formed typical clumps on the smear.

### Statistical analysis

Statistical comparisons between subgroups were performed using the chi-square test. Event-free survival was calculated according to the method of Kaplan and Meier. End points were the date of the first event, i.e. progression or death, and the date of the last follow-up evaluation when no event occurred (Kaplan and Meier, 1958). Curves were compared using the log-rank test.

Multivariate analysis was performed using the Cox proportional hazards model (Mantel and Haenzel, 1959). All statistical analyses were performed according to the procedure of the BMDP package (BMDP Statistical Software, Los Angeles, CA, USA).

### RESULTS

### TRK glycoprotein expression and its correlation with disease stage, age of the children, CD44 expression and N-*MYC* amplification

The expression of TRK glycoprotein was studied by immunochemistry on 122 tumour samples. Fourteen samples were classified as ganglioneuromas. TRKA expression was observed on the only two samples that contained ganglion cells and was restricted to this subpopulation, whereas Schwann cells were negative. Ganglioneuromas, which are known to be benign tumours, were excluded from the rest of the analysis.

The other 108 samples were classified as neuroblastomas or ganglioneuroblastomas. A positive immunostaining was observed on 60 of the 92 neuroblastomas and 14 of the 16 ganglioneuroblastomas.

A strong expression of TRKA was detected on the 12 stage IVS neuroblastomas and in most low-stage neuroblastomas (stage I, II and III) (44/50), whereas it was present on only 18 of 46 stage IV specimens. Therefore, *TRK* proto-oncogene expression was strongly related to the stage of the disease ($P < 10^{-5}$, $\chi^2$=29.75). Similarly, TRK expression was strongly correlated with the youth of the patients, since TRK was found in all but two tumours from infants, and in only 29 of 61 tumours from patients older than 1 year ($P < 10^{-5}$, $\chi^2 = 26.40$).

The expression of TRKA also correlated with the two other biological markers, i.e. N-*MYC* and CD44. Among the 108 analysed tumour samples, N-*MYC* amplification was defined for 105 specimens. Within the 25 tumours with N-*MYC* amplification, only eight expressed TRKA, whereas 17 did not. In contrast, within the 80 non-amplified samples, 63 expressed the TRKA proto-oncogene ($P = 4 \times 10^{-5}$, $\chi^2 = 16.94$). Seventy of 85 CD44-positive samples expressed TRKA, whereas only four of 23 CD44-negative specimens were TRKA positive ($P < 10^{-5}$, $\chi^2 = 32.47$).

### Event-free survival according to NMYC amplification, TRK and CD44 expression

The expression of TRKA strongly correlated with survival ($P = 10^{-6}$, LR = 28.6). Event-free survival at 3 years reached 81% in the group of patients with TRKA-positive tumours, whereas it was only 27% in TRKA-negative patients. When event-free survival was analysed according to the N-*MYC* status of the tumour, the survival rate at 3 years was 78% in the group of patients with non-amplified tumours vs 17% in patients with NMYC amplification. When event-free survival was analysed according to CD44 expression, the survival rate at 3 years was 78% in the group of patients with CD44-positive tumours vs only 13% in patients with tumours that did not express CD44.

Figure 1A shows survival according to patterns of both TRK expression and N-*MYC* amplification. The combination of the two markers allowed us to distinguish between patients with different prognosis: patients with TRK-positive and non-N-*MYC*-amplified tumours with 88% event-free survival at 3 years, patients with NMYC amplification and absence of TRKA expression with 10% event-free survival and an intermediary group of 25 patients with only one of the two abnormalities, i.e. NMYC amplification or TRK negativity (40% event-free survival in 17 patients with

© Cancer Research Campaign 1997

**Figure 1** Event-free survival in 108 patients. Combined analysis according to TRKA expression and NMYC amplification (**A**). In patients without N-*MYC* amplification, event-free survival in patients with or without TRKA expression was significantly different (LR = 13.7, $P < 10^{-3}$), whereas no difference was observed in the group of patients with NMYC amplification. Combined analysis according to TRKA expression and CD44 expression (**B**). In patients with CD44-positive tumours, event-free survival in patients with or without TRKA expression was significantly different (LR = 10.8, $P < 10^{-3}$), whereas no difference was observed in the group of patients with CD44-negative tumours, but the number of patients is too small to conclude that TRKA expression is not of prognostic value. NE, not evaluable; CI, confidence interval

TRKA-negative and N-*MYC* non-amplified tumours, and 32% event-free survival in the other eight patients with N-*MYC*-amplified tumour and TRKA expression).

Figure 1B shows survival according to the pattern of TRKA and CD44 expression. Within the group of patients with CD44-positive tumours, the expression of TRKA permitted us to distinguish between two groups with significantly different survival, i.e. patients with TRKA-positive tumours reached 86% event-free survival at 3 years, although those with TRKA-negative tumours only reached 38%. Survival at 3 years was less than 15% in patients with CD44-negative tumours, independently of the expression of TRKA, but the number of patients is too small to conclude that TRKA expression is not of prognostic value.

### Univariate and multivariate analyses of clinical and laboratory variables according to survival

We analysed the effect on survival of the expression of TRKA compared with other clinical and biological prognostic factors: patient's age, tumour stage, tumour histology, CD44 expression and N-*MYC* amplification (Table 1). On the basis of a univariate analysis, all factors had significant value, but CD44 expression, disease stage, absence of N-*MYC* amplification and TRKA expression were the most powerful predictors of a favourable outcome. In a stage-related analysis of event-free survival, the absence of N-*MYC* amplification, CD44 and TRKA expression were still predictors of favourable outcome in the group of patients with stage I–II, III and IVS disease ($P<10^{-3}$; $P=10^{-6}$; $P<10^{-4}$ respectively); in stage IV patients, N-*MYC* and CD44 remained significant ($P=0.02$; $P<0.02$), but TRKA had no prognostic value ($P=0.21$). In a multivariate analysis taking into consideration the prognostic factors previously mentioned, CD44 expression and tumour stage were the only independent prognostic factors for the prediction of patients' event-free survival.

**Table 1** Univariate and multivariate analysis of survival according to clinical and biological variables in patients with neuroblastoma

| | Univariate analysis | | Multivariate analysis | |
|---|---|---|---|---|
| | LR | P | LR | P |
| CD44 expression | 39.9 | $< 10^{-6}$ | 7.98 | 0.005 |
| N-*MYC* amplification | 32.3 | $< 10^{-6}$ | 1.11 | 0.293 |
| TRKA expression | 28.6 | $10^{-6}$ | 2.28 | 0.131 |
| Age < 12 months | 11.6 | $<10^{-3}$ | 0.49 | 0.484 |
| Stage | 33.04 | $<10^{-6}$ | 9.99 | 0.002 |
| Tumour histology | 5.3 | <0.005 | 2.39 | 0.122 |

The univariate analysis was performed on 108 patients, except for the evaluation of input of NMYC amplification that was known in only 105 patients. As a consequence, the multivariate analysis was performed on these 105 patients.

### DISCUSSION AND CONCLUSION

Previous studies have reported that a high level of expression of the TRKA mRNA in neuroblastoma is strongly predictive of a favourable outcome (Nakagawara et al, 1993; Kogner et al, 1993; Susuki et al, 1993; Borrello et al, 1993). Up to now, the analysis of TRKA by immunohistological methods suffered from a weak definition of reagent specificity and the absence of evaluation of its prognostic value. The current study was designed to determine whether the immunohistochemical expression of the *TRKA* gene had a similar clinical relevance and compare its value with that of already defined parameters, such as N-*MYC* amplification and CD44 expression. Thus, the polyclonal antibody that was used is specific for TRKA and does not show any cross-reactivity for TRKB or TRKC. During the preparation of this manuscript, Tanaka et al (1995) reported the immunohistological expression of TRKA on 105 neuroblastomas using the same reagent and came to the same conclusion. Unlike Northern blot, the analysis of TRKA by immunohistochemistry is a simple and reliable technique for use in routine clinical study; furthermore, it allows the study of samples, such as bone marrow aspirates or ultrasound-guided punctures of the primary tumour that contain only small quantities of tumour cells, or partially differentiated and well-differentiated tumours because TRKA expression is determined at the cell level on individual cells. For instance, the selective expression of TRKA on ganglion cells in ganglioneuromas and the negativity of Schwann cells were also observed by Donovan et al (1993) with an immunohistochemical method and might explain that these fully differentiated tumours express TRKA variably when studied at RNA level. As previously described for high levels of TRKA mRNA, TRKA protein expression is associated with young age at diagnosis, low clinical stage, absence of N-*MYC* amplification and

© Cancer Research Campaign 1997

favourable outcome of the patients. In addition, the expression of CD44 and that of TRKA are correlated and the combined analyses of TRKA expression and N-*MYC* amplification or CD44 expression permit the characterization of subgroups of patients with different prognoses.

The International Neuroblastoma Staging System and Response Criteria Committee recommend the study of biological features at the time of diagnosis in the prediction of prognosis and for the choice of the more appropriate therapeutic strategies. Different cellular and molecular markers have been determined, but the definition of the most useful markers to be used in a clinical setting suffers from a lack of comparison in multivariate analysis. In our study, four biological features of the tumour (histology, N-*MYC* copy number, CD44 expression and TRKA expression) could be analysed concomitantly. In a univariate analysis, these four parameters had significant value but, in a multivariate analysis, CD44 expression and disease stage were the only independent prognostic factors.

In conclusion, the immunohistological detection of TRKA is a strong prognostic factor in neuroblastoma when combined with N-*MYC* or CD44 assessment. In particular, the absence of TRKA expression on CD44-positive or N-*MYC* non-amplified tumours permits the characterization of a group of patients with intermediate prognosis. However, in a multivariate analysis, only CD44 and disease stage are independent prognostic factors. The development of simple and reliable micromethods, such as immunochemistry, allows the determination of different parameters on the same samples in routine examination. Optimally, these methods will enable comparison of the prognostic values of the different biological and clinical parameters in a multicentred prospective study and confirmation of the current data.

### ACKNOWLEDGEMENTS

We thank the following clinicians for providing tumour samples: A Deville, P Lutz, C Berger, P Wacker, A Signer and A Feldges. This work was supported by grants from the ARC (French Association for Cancer Research), the Fondation de France, the Rhône Committee of the French National League against Cancer, the Fédération Nationale des GEFLUC, the Swiss National Scientific Foundation (grant 3200-037544.93) and the Fondation FORCE (Fondation pour la Recherche du Cancer de l'Enfant).

### REFERENCES

Borrello MG, Bongarzone I, Pierotti MA, Luksch R, Gasparini M, Collini P, Pilotti S, Rizzetti MG, Mondellini P, De Bernardi B, Di Martino D, Garaventa A, Brisigotti M and Tonini GP (1993) *Trk* and *ret* proto-oncogene expression in human neuroblastoma specimens: high frequency of trk expression in non-advanced stages. *Int J Cancer* **54**: 540–545

Brodeur GM, Azar C, Brother M, Hiemstra J, Kaufman B, Marshall H, Moley J, Nakagawara A, Saylors R, Scavarda N, Schneider S, Wasson J, White P, Seeger R, Look T and Castleberry R (1992) Neuroblastoma. Effect of genetic factors on prognosis and treatment. *Cancer* **70**: 1685–1694

Combaret V, Wang Q, Favrot MC, Thiesse P, Philip I, Bouffet E, Bailly C, Bouvier R, Chauvin F, Zucker JM, Bernard JL, Lenoir G and Philip T (1989) Clinical value of N-*myc* oncogene amplification in 52 patients with neuroblastoma included in recent therapeutic protocols. *Eur J Cancer Clin Oncol* **24**: 1607–1612

Combaret V, Gross N, Lasset C, Frappaz D, Peruisseau G, Philip T, Beck D and Favrot MC (1996) Clinical relevance of CD44 cell surface expression and N*myc* gene amplification in a multicentric analysis of 121 pediatric neuroblastomas. *J Clin Oncol* **14**: 25–34

Donovan MJ, Hempstead BL, Horvath C, Chao MV and Schofield D (1993) Immunohistochemical localization of Trk receptor protein in pediatric small round blue cell tumors. *Am J Pathol* **143**: 1560–1567

Evans AE, D'Angio GJ and Randolph J (1971) A proposed staging for children with neuroblastoma. *Cancer* **27**: 374–378

Favrot MC, Combaret V, Goillot E, Lutz P, Frappaz D, Thiesse P, Thyss A, Dolbeau D, Bouffet E, Tabone E and Philip T (1991) Expression of integrin receptors on 45 clinical neuroblastoma specimens. *Int J Cancer* **49**: 347–355

Favrot MC, Lasset C and Combaret V (1993) CD44: a new prognostic marker for neuroblastoma. *N Engl J Med* **329**: 1965

Gross N, Beretta C, Peruisseau G, Jackson D, Simmons D and Beck D (1994) CD44H expression by human neuroblastoma cells: relation to MYCN amplification and lineage differentiation. *Cancer Res* **54**: 4238–4242

Kaplan EL and Meier P (1958) Nonparametric estimation from incomplete observations. *J Am Stat Assoc* **53**: 457–481

Kogner P, Barbany G, Dominici C, Castello MA, Raschella G and Persson H (1993) Coexpression of messenger RNA for TRK protooncogene and low affinity nerve growth factor receptor in neuroblastoma with favorable prognosis. *Cancer Res* **53**: 2044–2050

Mantel H and Haenzel W (1959) Statistical aspects of the analysis of data from retrospective studies of disease. *J Natl Cancer Inst* **22**: 719–748

Nakagawara A, Arima-Nakagawara M, Scavarda NJ, Azar CG, Cantor AB and Brodeur GM (1993) Association between high levels of expression of the *TRK* gene and favorable outcome in human neuroblastoma. *N Engl J Med* **328**: 847–854

Philip T, Bernard JL, Zucker JM, Pinkerton R, Lutz P, Bordigoni P, Plouvier E, Robert A, Carton R, Philippe N, Philip I, Chauvin F and Favrot M (1987) High dose chemotherapy with bone marrow transplantation as consolidation treatment in neuroblastoma: an unselected group of stage IV patients over one year of age. *J Clin Oncol* **5**: 266–271

Shimada H, Chatten J, Newton WA, Sachs N, Hamoudi AB, Chiba T, Marsden HB and Misuki K (1984) Histopathologic prognostic factors in neuroblastic tumors: definition of subtypes of ganglioneuroblastomas and age-linked classification of neuroblastoma. *J Natl Cancer Inst* **73**: 405–416

Susuki T, Bogenmann E, Shimada H, Stram D and Seeger RC (1993) Lack of high-affinity nerve growth factor receptors in aggressive neuroblastomas. *J Natl Cancer Inst* **85**: 377–384

Tanaka T, Hiyama E, Sugimoto T, Sawada T, Tanabe M and Ida N (1995) TrkA gene expression in neuroblastoma. *Cancer* **76**: 1086–1095

Zucker JM, Philip T, Bernard JL, Michon J, Bouffet E, Gentet JC, Lopez M, Coze C, Philip I, Bordigoni P, Plouvier E, Mazingue F and Vilcoq JR (1991) Single or double consolidation treatment according to remission status after initial therapy in metastatic neuroblastoma: first results of LMCE3 study in 40 patients. In *Advances in Neuroblastoma Research*, Evans A E, D'Angio G J, Knudson A G J and Seeder R C (eds), pp. 543–551. Wiley-Liss: New York

© Cancer Research Campaign 1997